# Virtual IP-based Secure Gatekeeper System for Internet of Things

**DOI:** 10.3390/s21010038

**Published:** 2020-12-23

**Authors:** Younchan Jung, Ronnel Agulto

**Affiliations:** School of Information, Communications, and Electronics Engineering, The Catholic University of Korea, Seoul 07345, Korea; ronnelagulto@catholic.ac.kr

**Keywords:** gatekeeper, virtual IP address, one-time AES key, mobile edge effects, DNS

## Abstract

The advantage of using the Network Address Translation device is that the internal IP address, which makes the IP address space of Internet of Things (IoT) devices expanded, is invisible from the outside and safe from external attacks. However, the use of these private IPv4 addresses poses traversal problems, especially for the mobile IoTs to operate peer-to-peer applications. An alternative solution is to use IPv6 technologies for future IoT devices. However, IPv6 package, including IPSec, is too complex to apply to the IoT device because it is a technology developed for the user terminal with enough computing power. This paper proposes a gatekeeper to enable the real IP addresses of IoTs inside the same subnetwork to be not explicitly addressable and visible from outside of the gatekeeper. Each IoT device publishes its virtual IP address via the Registrar Server or Domain Name System (DNS) with which the gatekeeper shares the address mapping information. While the gatekeeper maintains the mapping information for the local IoT devices, the registration server or DNS has global address mapping information so that any peer can reach the mapping information. All incoming and outgoing packets must pass through the gatekeeper responsible for the address conversion and security checks for them from the entrance. This paper aims to apply our gatekeeper system to a platform of self-driving cars that allows surrounding IoT cameras and autonomous vehicles to communicate with each other securely, safely, and rapidly. So, this paper finally analyzes improvement effects on latency to show that our gatekeeper system guarantees the latency goal of 20 ms under the environment of 5G links.

## 1. Introduction

Modern smart cities still need to allow IPv4 network-connected Internet of Things (IoT) devices. The Network Address Translation (NAT) was originally invented to address the shortage of IPv4 address space. Also, NAT has been used to hide the network and hosts from the external network. The advantage of using the Network Address Translation (NAT) device is that the internal IP address, which makes the IP address space expanded, is invisible from the outside and safe from external attacks [1]. However, the use of this private IPv4 addresses poses traversal problems, especially for the mobile IoTs to operate peer-to-peer applications [2,3,4,5]. Conventional NAT devices enable end nodes inside local networks to be not explicitly addressable and visible. NAT’s motivation is that all devices in the same local network can change their addresses and use just one IP address (NATed address), notifying it to the outside world.

Unlike the NAT approach, each device in the same domain can use a different virtual IP address to be safe from external attacks. While NATed end nodes use the same NATed IP address, the real address can be mapped to a random virtual address visible to the outside world. There are two existing directions connected with the virtual IP address: Moving Target Defense (MTD) technique and the load balancing technique in a massive server farm. One of the MTD techniques has been proposed for a Software-Defined Networking (SDN) based in-vehicle network architecture [6]. When an IP packet from an Electronic Control Unit (ECU) node comes to the SDN switch, the SDN controller replaces the IP address with a random virtual address, and then the virtual address is used for forwarding the packet. In the last SDN switch, the virtual address is converted to the original address and sent to the destination ECU node [7]. Another proposed SDN-based MTD technique aims to defend against network reconnaissance and scanning attacks. This technique enables a host machine to have multiple, random, time-varying virtual IP addresses. The virtual IP address is useful so that server load balancers can scale easily to meet a massive server farm’s demands as the amount of traffic and the number of servers in the server farm grows. Server load balancers can be scalable if the incoming data traffic to the gateway router at the data center is forwarded according to the assigned virtual IP address [8].

Lightweight Machine-to-Machine (LwM2M) is a protocol for M2M or IoT device management and service enablement. The LwM2M standard defines the application layer communication protocol between an LwM2M server and an LwM2M client located in an IoT device. It offers an approach for managing IoT devices and allows devices and systems from different vendors to co-exist in an IoT- ecosystem. LwM2M’s device management capabilities include remote provisioning of security credentials, firmware updates, connectivity management, remote device diagnostics, and troubleshooting. Meanwhile, the peer-to-peer (P2P) model for IoT devices is contrasted to the LwM2M client-server architecture. Either IoT sensors in the local area, which have its dedicated spectrum/LAN managed by a local gateway, or the IoT applications, which cover areas that span a large geographic area, need their IP addresses, which are easy to be caught by an external attacker. This paper’s first goal is to suggest the dual use of the virtual IP address and real IP address for them. This paper proposes the gatekeeper to enable the real IP addresses of IoTs inside local subnetworks to be not explicitly addressable and visible from outside of the gatekeeper. The IoT device publishes its virtual IP address via the Registrar Server or Domain Name System (DNS). The IoT device, gatekeeper, and Registrar Server (or DNS) share information about the relationship between the real IP address and virtual IP address [9,10,11,12,13,14,15]. The gatekeeper maintains an Address Mapping Table (AMT) in which the AMT matching number indicates each entry describing that relationship.

All incoming and outgoing packets must pass through the gatekeeper as an entrance and exit gate. This paper pursues that all of the world’s IoT devices can be associated with their distributed gatekeepers. Then, the gatekeeper can protect its IoT devices from the traffic analysis attacks that try to scan the IoT devices from the outside and analyze the role of them [16,17,18,19]. Also, because the gatekeeper is responsible for security checks at the entrance, the low-end IoT device inside the gatekeeper is free from the burden to check whether the incoming packet is malicious. On behalf of the IoT device, the gatekeeper protects this malicious packet’s intrusion from the entrance [20].

IoT devices can use IPv6 technology to collaborate with the gatekeeper to use dual IP addresses without using NAT devices. In the gatekeeper system with IPv6, 128-bit address conversion occurs, and 128-bit mapping information needs to be handled in the registrar server or DNS. To deal with 128-bit address information affects to increase the complexity of the gatekeeper system. Also, the IPv6 package is too expensive to deploy, leaving local networks ill-equipped with the mobility and security needed in a reliable IoT environment [21,22]. Internet Protocol Security (IPSec) is a secure network protocol suite that authenticates and encrypts data packets to secure communication between two computers over an Internet Protocol (IP) network. It is often used in virtual private networks (VPNs). IPSec technology can be a solution for the IoT devices to handle end-to-end security management within the network layer (layer 3) [23,24]. In particular, IETF recommends using IPv6 as a layer three protocol of the IoTs where the use of IPSec is mandatory when using IPv6. As IPSec’s main features, IoTs using IPv6 should manage the Security Policy Database and Security Association management to indicate security algorithms and secret keys to use. Therefore, IPsec technology is too complex to apply to the IoT device because it is a technology developed for the user terminal with enough computing power [25]. Although there is a lot of existing efforts concerning the design of lightweight asymmetric key systems, asymmetric key systems (i.e., public/private keys) still take too many computing resources for low-end IoT hardware [26,27,28,29,30]. Therefore, it is straightforward that the SSL (Secure Socket Layer)/TLS (Transport Layer Security) cannot be applied to handle end-to-end security processing for the IoT [31,32].

In the application layer, the symmetric key system can provide confidentiality services to protect the IoT device. The key here is to share the symmetric key between the two end nodes. The Diffie-Hellman (DH) key exchange algorithm can be a solution. DH key exchange is a method of securely exchanging cryptographic keys over a public channel. DH is one of the earliest practical examples of public key exchange implemented within the field of cryptography. Diffie–Hellman key agreement itself is a non-authenticated key-agreement protocol. Usually, the DH algorithm is vulnerable to man-in-the-middle (MITM) attacks when exchanging DH public values between two devices. A third party can intervene in the middle and manipulate the DH public value exchanged with each other. Therefore, the DH algorithm requires additional complementary measures for authentication [33].

Secondly, the contribution of this paper is the introduction of the gatekeeper, which can support end-to-end secure sessions between two IoTs located behind their gatekeepers. Security concerns regarding the confidentiality and authentication of the peers’ communication are solved using some gatekeeper-made security scheme. In the paper, the DH public value of an IoT device also functions as its gatekeeper’s AMT matching number. If an attack on the DH public value during the DH key exchange phase occurs, [virtual IP address/real IP address] (or [real IP address/virtual IP address]) conversion in the gatekeeper does not work properly. As a result, mutual agreement on the DH key will fail. Therefore, the gatekeeper exerts the effect of thoroughly blocking the MITM attack in the DH key exchange.

The third contribution of this paper relates to using edge computing at a secure gatekeeper. Ensuring the reliability of the in/out messages for the low-end IoT device of limited capacity is possible if the server as a third party involves confirming the message’s maliciousness. Our gatekeeper at the local network entrance acts as the third-party server. Then, the communication time delay, which occurs in Vehicle-to-Vehicle (V2V) communication via a gatekeeper system, can be similar to the direct device-to-device (D2D) communication level. This idea is similar to the 5G system to move computing smarts from the central servers to the 5G base stations. Communication of latency-critical IoT applications requires a latency level of somewhere between 1 and 100 ms [34,35,36]. Self-driving vehicles can prevent sudden accidents efficiently if the cars and near IoT cameras collaborate to exchange the time-sensitive information in real-time [37]. IoT devices, such as vehicles or IoT camera devices that operate in a local area, are located within a sub-network in the self-driving environment. At the entrance of the sub-network, their gatekeeper relays the incoming and outgoing packets between them. The autonomous vehicle needs instant information from nearby IoT devices such as IoT cameras that leave the sight with minimum latency in real-time. The latency goal of our secure gatekeeper system is to reduce device-to-device communication latency so that the V2V, IoT-to-Vehicle (IoT2V) or Vehicle-to-IoT (V2IoT) communication satisfies the latency required to the level of 20 ms. Suppose a car driving through the city at 60 km/h receives information about an emergent situation within 60 ms and copes with the situation. In that case, it can react within a moving distance of about 1 m away. This paper aims to limit the IoT2V data transfer latency over the 5G networks within 20 ms, making the reaction possible within a moving distance of about 0.3 m away.

**Figure 1:**
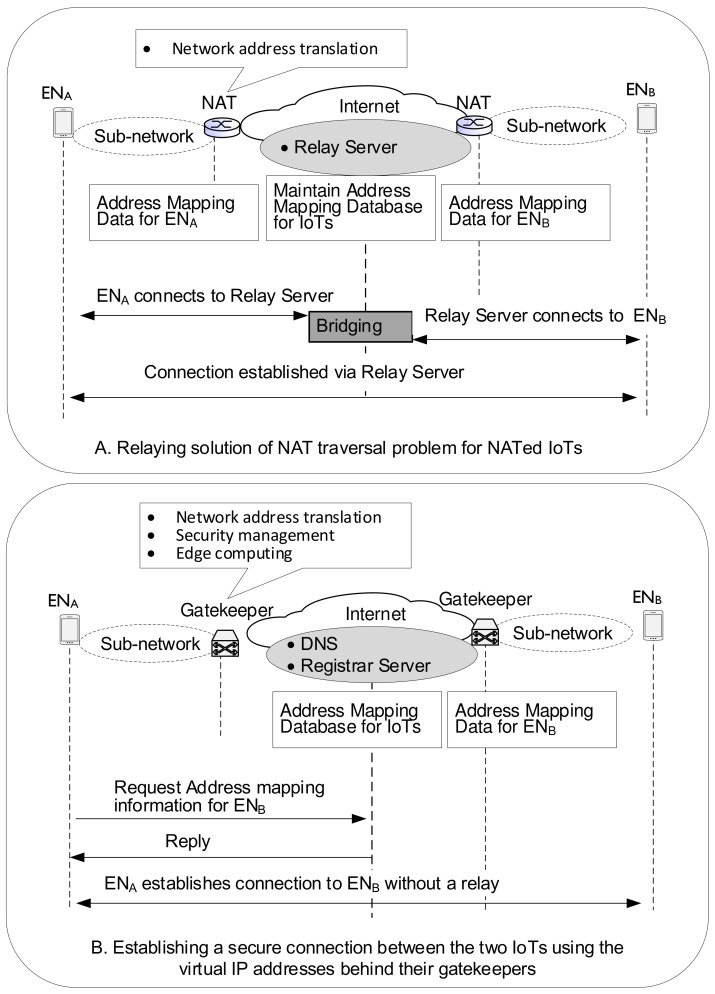
Comparison of NAT and Gatekeeper approaches that use invisible IP addresses from the outside.

Figure 1 shows a comparison of NAT and gatekeeper approaches that use invisible IP addresses of end nodes (ENs) from the outside. NAT operations are simple. Without any collaborations with other systems, the NAT device decides to map the IP address and port number of packets in the outgoing direction to the new IP address and port number exposed to the outside world. Regarding the incoming packets, the NAT executes the address and port number conversions by referencing this mapping information created by itself. The relay server in the NAT system has to know the mapping information needed to convert network addresses. In other words, the NAT device is only an executor to act. However, the gatekeeper shares the address mapping information with the registration server or DNS. While the registration server or DNS has global address mapping information, the gatekeeper maintains the mapping information for the local IoT devices. Also, the gatekeeper shares the information necessary for security management with the registration server or DNS. While the NAT system requires other completely different methods to deal with security management or edge computing, the gatekeeper holds security management information. It also leads edge computing for its own IoT devices. Therefore, the gatekeeper approach enables moving the necessary computing for network address translation and security management from the central servers to the gatekeepers located at the subnetwork entrances. The use of gatekeepers is advantageous. For example, communication between two IoT devices connected to the same subnet using an identical gatekeeper occurs via the gatekeeper. However, an external relay server must be involved even though two adjacent devices behind the same NAT can exchange data. Similarly, from a security management perspective, gatekeepers can perform security management without external servers’ assistance. The NAT system’s relay server, which is entirely responsible for solving traversal problems, is usually installed and used privately for commercial purposes for the NAT-related applications. It is a relay server that is generally used at the top of the commercial vertical model. On the other hand, the registration server or DNS as a horizontal model center provides public services to ensure that the distributed gatekeepers operate independently.

The rest of this paper is organized as follows. Section 2 explains the secure gatekeeper-centric approaches for the use of virtual addresses. In Section 3, this paper explains three different models of the secure gatekeeper system for Peer-to-Peer, HTTP, and IoT2V communication. Section 4 discusses about the improvement effects for the secure gatekeeper system. This paper concludes in Section 5.

## 2. Secure Gatekeeper-Centric Approaches Using Virtual Addresses

Real IP addresses inside the gatekeeper and private IP addresses inside the NAT devices are hidden from the outside. As shown in Figure 1, the relay server’s help is essential to solving the NAT traversal problem for the NATed IoTs. Compared with the existing NAT approach, the real difference is that the gatekeeper approach enables moving the necessary computing for network address translation and security management from any central servers to the gatekeepers located at the subnetwork entrances.

### 2.1. Existing NAT Devices to Hide the Actual Address

The application running on mobile IoTs connected to the Internet from various places will not have a fixed network address. Moreover, how to connect Peer-to-Peer (P2P) applications between two different inter-subnet IoTs is a concern. Those IoTs may connect to the Internet via a NAT device as long as NAT devices are used to bridge between IPv4 and full IPv6 networks. Here’s a scenario: an application requires TCP connections between two IoT devices that are both behind NAT devices. The problem is that both IoTs get private IP addresses and must send their communication to the public Internet through NAT devices. The NAT device uses port translation to determine which IoT should receive the responses. For example, when the NAT device receives communication from a private IP address and original port number, it sends the communication out via its public IP address on a different proxy port, which only itself knows. Then, the NAT maintains a table of mappings for the outbound packet stream. However, outside IoT will face difficulty getting knowledge about the destination NAT’s mapping information, which enables the destination NAT to deal with the inbound packets. Popular P2P applications have addressed this NAT Traversal Problem in different ways because the traversal problem is solved together with several issues related to security, mobility, and scalability.

NAT would work well for typical client/server communications since it’s always the client that initiates the communication session. However, in P2P, the media flow directly between the two peers. Then, each peer should obtain other peer’s public transport addresses instead of its private address to establish a session to traverse the intermediate NAT device. NAT devices, which operate in a distributed manner, don’t behave uniformly. So, for the P2P application designer, to solve the traversal problem is challenging.

Conventional NAT devices enable end nodes inside local networks to be not explicitly addressable and visible. NAT’s motivation is that all devices, which can change their addresses in the local network, use just one IP address (NATed address), notifying it to the outside world. The idea of NAT is to use a 16-bit port-number field to extend address space. So the use of the port-number field makes it possible to have about 60,000 simultaneous connections with a single NATed address. However, NAT is controversial because NAT devices should process up to layer four, while typical routers do up to layer 3. The NAT device keeps the NAT mapped table that enables incoming and outgoing datagrams to change their source port number and source IP address. So, the NAT mapped table containing the dynamically and locally changing database is difficult to understand in a peer-to-peer (P2P) network when a peer wants to initiate a new communication with another peer connected to the Internet via NAT. The most significant drawback is that it becomes challenging to track mutual IP addresses between two end-to-end nodes. When they want to set up a session, each side needs to obtain the other party’s NATed IP address and the internal IP address used inside the NAT device. However, if the path between them includes multiple NAT routers, it becomes more challenging to track their real IP addresses. If a local node operates with a NATed IP address, the other party cannot establish a successful session with that local node. Also, the NAT router scans layer 3 and 4 headers of passing packets, so the path’s switching delay becomes larger. Considering that Strict conditions on latency issues are required for a self-driving car that operates as one of the IoT nodes, it isn’t straightforward for self-driving vehicles to work behind NAT routers.

### 2.2. Prerequisites for Secure Gatekeeper Operations

The existing SIP (Session Initiation Protocol) phone system requires the registrar server to identify people by names and reach callee, no matter where callee roams, no matter what IP device callee is currently using. Our Registrar Server can include the application to provide the name resolution service for IoT devices as well as SIP phone users. At the same time, DNS is the application dedicated to the name resolution service. Then, both Registrar Server and DNS maintain resource records necessary to resolve name/real address mapping. Real IP addresses can be used to discern IoT physical location. These addresses also act like IoT identifiers, letting attackers track them online. However, our Registrar Server and DNS involve resolving name/virtual address mapping. The DNS (or Registrar Server) query/answer between two different IoT nodes enables the initiating IoT node to obtain the virtual address information corresponding to the questioned name. On the other hand, to fix the virtual address-real address relationship is the gatekeeper’s role. This paper does not consider L2 (Layer 2) connections established inside sub-network. Our gatekeeper always relays L3 packets from close IoT devices such as vehicles or IoT camera devices, which operate in a local area in the self-driving environment. Since L3 packets between close IoT devices must be exchanged via the gatekeeper, the gatekeeper can behave as a defense agent compensating for the point that IoT devices cannot directly block the malicious packet’s intrusion attacks due to their limited computing resources. The gatekeeper intervenes in the communication of all IoT devices and monitors whether the malicious packets appear in the IoT environment. This point is another reason why this paper aims to apply the gatekeeper system to V2V, IoT2V, or V2IoT communication. Because of the gatekeeper’s surveillance role, the cars can trust the nearby IoT device’s data and use it for autonomous driving. Then, there is no way to attack the self-driving car via an L2 connection for the inside attacker.

**Figure 2:**
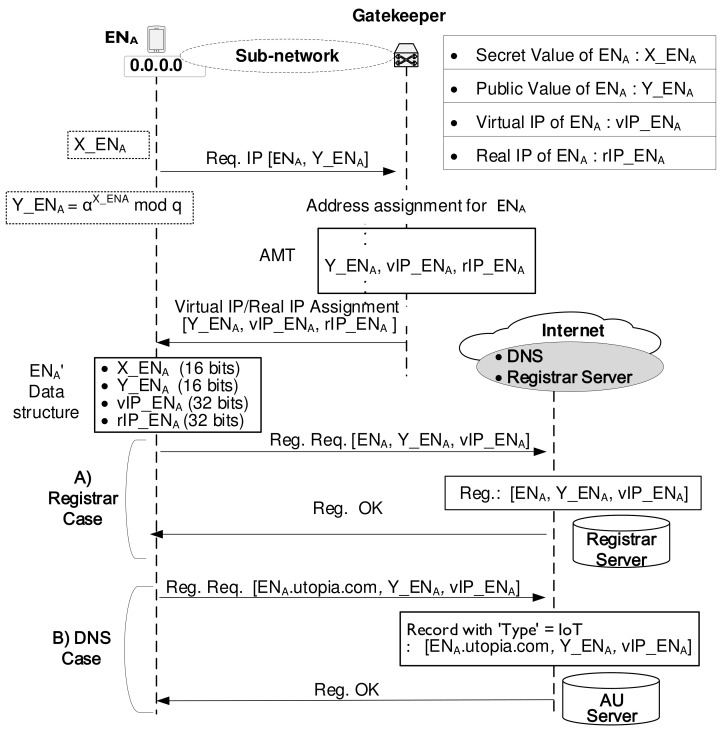
IoT device, gatekeeper, and Registrar Server (or DNS) sharing information about the relationship between the real IP address and virtual IP address.

Figure 2 shows how an IoT device (the name is $EN_{A}$, and the domain name is assumed to be $EN_{A}$ .utopia.com) obtains its address-specific information from the gatekeeper and register them on the Registrar Server or DNS. The initial registration procedure consists of 3 steps.

**STEP 1:** Suppose an end node (EN) wishes to operate behind some gatekeeper. Then, the EN selects a random integer, that is, secret value (X_EN) and computes corresponding blind value, that is, Y_EN where $Y\_EN=\alpha^{X\_EN}\;mod\;q$. In the DH cryptography system, a set of global public elements includes a large prime number of *q* and α integer, the primitive root of *q*. The size of secret keys can be any value within the range [1, q−1]. Assuming that each gatekeeper handles the maximum number of $2^{16}$ end nodes, the size of the prime number *q* needs to be as much as 16 bits. In the face of a need for a new IP address, EN requests IP addresses to the gatekeeper by sending its name and blind value, that is, EN and Y_EN.

**STEP 2:** Upon receiving this request, the gatekeeper assigns real IP address (rIP_EN) and virtual IP address (vIP_EN) for Y_EN, which constitutes a new entry in the Address Mapping Table (AMT). Then, the gatekeeper maintains the triple values for EN:

- 16-bit Y_EN,
- 32-bit rIP_EN,
- 32-bit vIP_EN.

The end node uses Y_EN as the DH cryptography value to compute the DH secret key while the gatekeeper considers it the AMT matching number to find the corresponding entry in the AMT. Upon receiving the above triple values from the gatekeeper, EN maintains the following data structure:

- X_EN,
- Y_EN,
- N_EN (a nonce of EN used for the security management),
- rIP_EN,
- vIP_EN.

**STEP 3:** As soon as EN acquires its rIP_EN and vIP_EN, it registers this allocated information directly with the Registrar Server or the AUthoritative (AU) server of the DNS system. Therefore, the IoT device, gatekeeper, and Registrar Server (or DNS) share information about the relationship between the real IP address and virtual IP address.

### 2.3. Proposed Secure Gatekeeper

The gatekeeper involves allocating address-specific information as a response of the end node’s request and plays the agent role of changing the header addresses for packets traveling through the gatekeeper. The gatekeeper maintains the Address Mapping Tables (AMT). Each entry in the AMT contains mapping information of [*Y*, vIP, rIP], where the blind value of *Y* points a specific entry. When a datagram from the end node passes through the gatekeeper, it only handles the layer three header fields. For the outgoing datagram and incoming datagram, the gatekeeper performs rIP-vIP and vIP-rIP translations.

**Figure 3:**
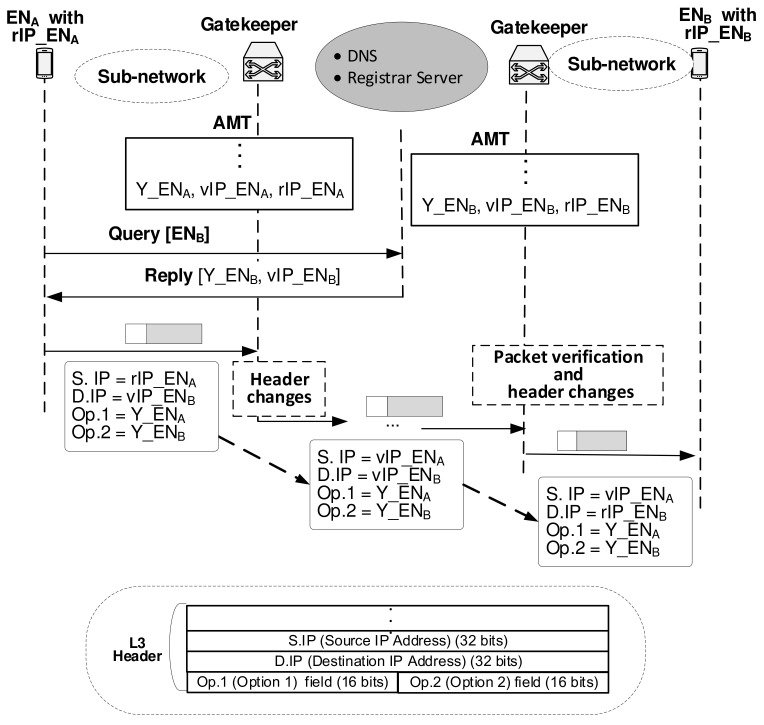
Verification of passing packets and IP header conversion of passing packets through the gatekeeper.

As indicated in Figure 3, when the calling device $EN_{A}$ wants to communicate with the called device $EN_{B}$, $EN_{A}$ should identify [AMT matching number, virtual IP address] of the $EN_{B}$. Then, $EN_{A}$ begins a Query/Reply procedure toward a communication session control system selected by the device. After $EN_{A}$ obtains the [$Y\_{EN}_{B}$, $vIP\_EN_{B}$] information of $EN_{B}$, $EN_{A}$ can send a packet to the destination of $EN_{B}$. When $EN_{A}$ sends the packet, all four fields of the packet header related to the address area are:

- 32-bit source IP address (S.IP),
- 32-bit destination IP address (D.IP),
- 16-bit option 1 (Op.1), which is the $EN_{A}$-side gatekeeper’s AMT matching number for $EN_{A}$ and
- 16-bit option 2 (Op.2), which is the $EN_{B}$-side gatekeeper’s AMT matching number for $EN_{B}$.

$EN_{A}$ sends the packet with the header of [S.IP = $rIP\_EN_{A}$, D.IP = $vIP\_EN_{B}$, Op.1 = $Y\_{EN}_{A}$, Op.2 = $Y\_{EN}_{B}$]. When the packet reaches the $EN_{A}$-side gatekeeper, it replaces the packet header with [S.IP = $vIP\_EN_{A}$, D.IP = $vIP\_EN_{B}$, Op.1 = $Y\_{EN}_{A}$, Op.2 = $Y\_{EN}_{B}$], and sends it out to the direction of the final destination. When the packet reaches the $EN_{B}$-side gatekeeper by conventionally passing through the internet, the gatekeeper ensures the packet is qualified to pass. That is, it checks whether the field of Op.2 of the packet matches the proper entry in the AMT. Recall that the field of Op.2 contains a 16-bit blind value of $Y\_{EN}_{B}$, which was answered by the Registrar Server or the AUthoritative (AU) server of the DNS. Because they certify the 16-bit blind value, only for the packet with certified blind value, the gatekeeper replaces the packet’s header with [S.IP = $vIP\_EN_{A}$, D.IP = $rIP\_EN_{B}$, Op.1 = $Y\_{EN}_{A}$, Op.2 = $Y\_{EN}_{B}$]. The verified packet reaches $EN_{B}$ with the destination IP address of $rIP\_EN_{B}$.

### 2.4. Security Management

The simplest and the original implementation of the DH protocol uses the multiplicative group of integers modulo *q*, where *q* is prime, and α is a primitive root modulo *q*. These two values are chosen in this way to ensure that the resulting shared secret can take on any value from 1 to q−1. On a mathematical level, the DH key exchange relies on one-way functions as the basis for its security. That is, ${EN}_{B}$-side DH key ($DHK_{B,A}$) is calculated as ${Y\_{EN}_{A}}^{X\_{EN}_{B}}\;mod\;q$ where $Y\_{EN}_{A}$ = $\alpha^{X\_{EN}_{A}}\;mod\;q$. These are calculations that are simple to do one way but much more difficult to calculate in reverse. Here, only $X\_{EN}_{B}$ and $X\_{EN}_{A}$ are kept secret. All the other values are sent in the clear. The strength of the scheme comes from the fact that $\alpha^{X\_{EN}_{A}\cdot X\_{EN}_{B}}\;mod\;q$ = $\alpha^{X\_{EN}_{B}\cdot X\_{EN}_{A}}\;mod\;q$ take extremely long times to compute just from the knowledge of *q*, α, $\alpha^{X\_{EN}_{B}}\;mod\;q$, and $\alpha^{X\_{EN}_{A}}\;mod\;q$. Such a problem is called the discrete logarithm problem. On the other hand, the computation of $\alpha^{X\_{EN}_{B}}\;mod\;q$ is known as modular exponentiation and can be done efficiently even for large numbers. Once ${EN}_{A}$ and ${EN}_{B}$ compute the shared secret, they can use it as an encryption key, known only to them, for sending messages across the same open communications channel.

The Diffie–Hellman key exchange method allows two parties with no prior knowledge of each other to establish a shared secret key over an insecure channel. This key can then be used to encrypt subsequent communications using a symmetric key cipher. Basic Diffie–Hellman key agreement itself is a non-authenticated key-agreement protocol. Usually, the DH algorithm is vulnerable to MITM attacks when exchanging public values between the two devices. It is possible that a third party intervenes in the middle and extracts the DH public value that each other exchanges. In our secure gatekeeper system, the Registrar Server (or DNS) stores the DH public values for the registered IoT devices, enabling the session initiator to obtain the other peer’s DH parameter on the shared key generation. A session responder can extract DH public value, which is contained in the Session Request message. Then, each side can share the same secret key as a function of the peer’s DH public value and its self-keeping secret value. Then, each end node requires to register its DH public value in the Registrar Server or DNS system.

Below we explain the gatekeeper-made authentication scheme. If any node can obtain the DH public value of the target node through Registrar Server, a malicious entity can also obtain it. When the malicious entity (A) uses it to reach the target node (B), A should present B’s DH value as a session initiator. That is, the session request packet should contain B’s DH value. When the packet arrives at the A-side gatekeeper, the gatekeeper will convert the A’s real IP address into its virtual IP address. If the entity is malicious, the gatekeeper fails to address conversion. So, the evil entity cannot succeed in sending out the session request through its source gatekeeper unless it uses its fair DH public value in which the Registrar server trusts. When the packet arrives at the B-side gatekeeper, the gatekeeper will convert the B’s virtual IP address into its real IP address. If B’s DH public value is modified, the gatekeeper fails to address conversion. So, the session request fails to pass through the destination gatekeeper if A attempts to use a DH public value different from B’s fair DH public value. This scheme is our gatekeeper-made authentication scheme.

**Figure 4:**
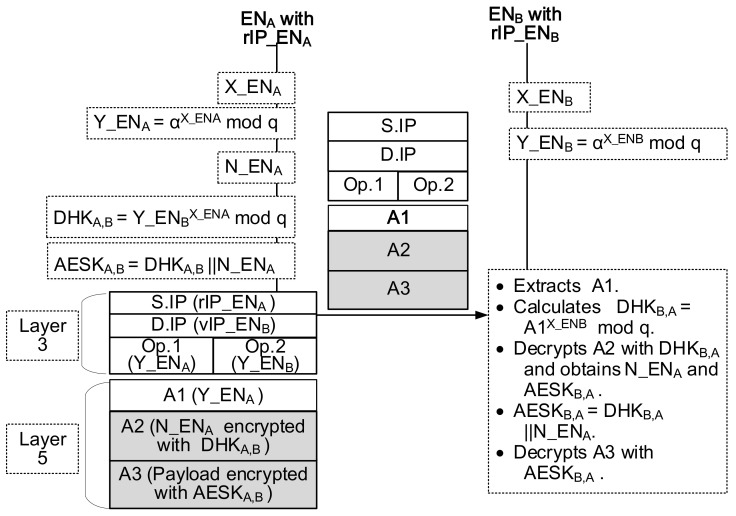
112-bit nonce, 16-bit DH key and 128-bit one-time AES key.

From security viewpoints, the session initiator node creates a nonce number on a session basis. The purpose of using the nonce value is to make the final symmetric key one-time session key and to adjust the size of the final symmetric key to 128 bits that can apply to the Advanced Encryption System (AES). As shown in Figure 4, ${EN}_{A}$ creates a nonce (112 bit-size $N\_{EN}_{A}$) and calculate the 16 bit-size DH key, that is, $DHK_{A,B}={Y\_{EN}_{B}}^{X\_{EN}_{A}}\;mod\;q$. And it concatenates the DH key and the nonce. This process results in 128 bit-size AES key, that is, $AESK_{A,B}=DHK_{A,B}\left|\right|N\_{EN}_{A}$. The application layer of the sending packet consists of the application header and payload. The application header contains the public value in a clear form ($Y\_{EN}_{A}$) and $N\_{EN}_{A}$ encrypted with $DHK_{A,B}$. The payload is encrypted with $AESK_{A,B}$. As a destination node, ${EN}_{B}$ receives the packet and extracts the first part of the application header and obtains $Y\_{EN}_{A}$. Next, ${EN}_{B}$ calculates the 16 bit-size DH key, that is, $DHK_{B,A}={Y\_{EN}_{A}}^{X\_{EN}_{B}}\;mod\;q$. And it decrypts the second part of the application header using the DH key of $DHK_{B,A}$ and obtains $N\_{EN}_{A}$. Now, ${EN}_{B}$ can produce its AES key $AESK_{B,A}$ by concatenating $DHK_{B,A}$ and $N\_{EN}_{A}$. As a result, ${EN}_{B}$ can decrypt the payload using $AESK_{B,A}$.

## 3. Secure Gatekeeper System

As the existing NAT approaches rely on the relay servers to solve the traversal problems, the EN, as the session initiator, needs to obtain the necessary parameters from external servers to pass the peer-side gatekeeper and carry out end-to-end security management. The secure gatekeeper system (SGS) can operate in three different ways to obtain those parameters: RS SGS (Registrar Server-based Secure Gatekeeper System), DNS SGS (DNS-based Secure Gatekeeper System), and MEC (Mobile Edge Computing) SGS. RS SGS and DNS SGS are for inter-gatekeeper communication, while MEC SGS is for intra-gatekeeper communication inside the sub-network. RS SGS and DNS SGS use Registrar Server and DNS to resolve the corresponding virtual IP address and DH public value for a given IoT name, respectively. MEC SGS effects of reducing latency, especially in the self-driving vehicle environment.

### 3.1. RS-Based Secure Gatekeeper System

**Figure 5:**
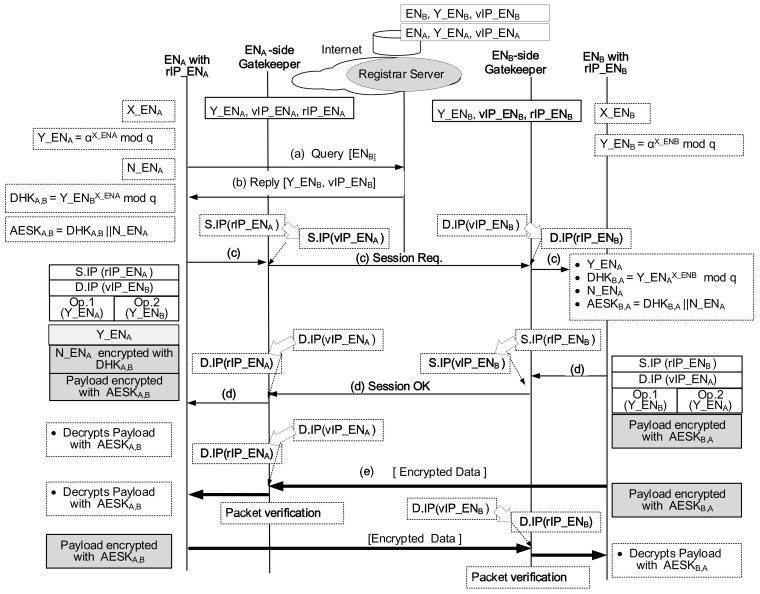
Secure peer-to-peer communication over RS-based Secure Gatekeeper System.

Table 1 shows three different modes of the SGS operations. Figure 5 shows the P2P (Peer-to-Peer) communication between the two IoT devices attached to the different gatekeepers. The calling device uses the query/response process to obtain the callee device’s DH public value and virtual IP address first through the Registrar Server. The name of the calling device is $EN_{A}$, and its gatekeeper keeps the AMT entry of [DH public value = $Y\_{EN}_{A}$, virtual IP address = $vIP\_EN_{A}$, real IP address = $rIP\_EN_{A}$]. On the other hand, the name of the callee device is $EN_{B}$, and its gatekeeper maintains the AMT entry of [DH public value = $Y\_{EN}_{B}$, virtual IP address = $vIP\_EN_{B}$, real IP address = $rIP\_EN_{B}$]. Below we enumerate steps to open secure peer-to-peer communication over RS-based Secure Gatekeeper System.

- The calling device $EN_{A}$ sends (a) Query to the Registrar Server to obtain the public value and virtual IP address information of $EN_{B}$.
- After receiving this query, the Registrar Server searches $EN_{B}$’s public information [$Y\_{EN}_{B}$, $vIP\_EN_{B}$] from the database and sends (b) Reply to $EN_{A}$ in a response format.
- When $EN_{A}$ obtains the [$Y\_{EN}_{B}$, $vIP\_EN_{B}$] information of $EN_{B}$, finally, $EN_{A}$ tries to send a session request packet to the destination of $EN_{B}$. Using the disclosure value obtained in response ($Y\_{EN}_{B}$) and $EN_{A}$’s secret value ($X\_{EN}_{A}$), $EN_{A}$ calculates $EN_{A}$-side 128 bit-size AES key, that is, $AESK_{A,B}=DHK_{A,B}\left|\right|N\_{EN}_{A}$ where $DHK_{A,B}$ = ${Y\_{EN}_{B}}^{X\_{EN}_{A}}\;mod\;q$. For (c) Session Request (SReq) packet to be sent, [Op.1 = $Y\_{EN}_{A}$, Op.2 = $Y\_{EN}_{B}$] constitutes the packet header’s relevant fields concerning security. The application layer of the SReq packet consists of the application header and payload. The application header contains the public value in a clear form ($Y\_{EN}_{A}$) and $N\_{EN}_{A}$ encrypted with $DHK_{A,B}$. The payload is encrypted with $AESK_{A,B}$.
- When this encrypted packet arrives via the Internet at the gatekeeper belonging to $EN_{B}$, the gatekeeper examines the packet’s header field of [Op.2 = $Y\_{EN}_{B}$] and finds the actual IP address of $EN_{B}$. Here, a hacker cannot modify the public value of $Y\_{EN}_{B}$. If the value of $Y\_{EN}_{B}$ changes, the packet cannot be delivered to $EN_{B}$ correctly.
- When $EN_{B}$ receives the encrypted packet, it extracts the field value of [Op.1 = $Y\_{EN}_{A}$] and compares to the public value $Y\_{EN}_{A}$ of the beginning of the application header. If $EN_{B}$ confirms that these two values are the same, it considers this $Y\_{EN}_{A}$ of $EN_{A}$ to be safe from hacker attacks. With the extracted public value $Y\_{EN}_{A}$ information and the secret value $X\_{EN}_{B}$, $EN_{B}$ calculates the symmetric key $DHK_{B,A}$ as follows. $DHK_{B,A}$ = ${Y\_{EN}_{A}}^{X\_{EN}_{B}}\;mod\;q$. Then, $EN_{B}$ obtains $N\_{EN}_{A}$ by decrypting process using the DH key of $DHK_{B,A}$. The payload is decrypted with $AESK_{B,A}$ ($DHK_{B,A}\left|\right|N\_{EN}_{A}$). Finally, $EN_{B}$ sends (d) Session OK to agree to open a secure session.
- In the following two-way end-to-end data communication, encrypted data flow travels through the path over the two different gatekeepers.

### 3.2. DNS-Based Secure Gatekeeper System

The idea of a DNS-based Secure Gatekeeper System is motivated by the fact that the existing DNS system can determine the named IoT device’s address. However, the original HTTP protocol is not designed for IoT connections with remote locations where the network bandwidth is limited. Among majorly used application protocols in IoT, protocols such as IPv6 over Low power Wireless Personal Area Network (6LoWPAN) and Constrained Application Protocol (CoAP) provide a feature through which smart IoT objects can integrate into the IP-driven networks [38]. This paper considers some application protocols in IoT which can collaborate with existing DNS system and calls them as a kind of smart HTTP protocols where a small-sized control message can carry several tens of bytes of data.

**Figure 6:**
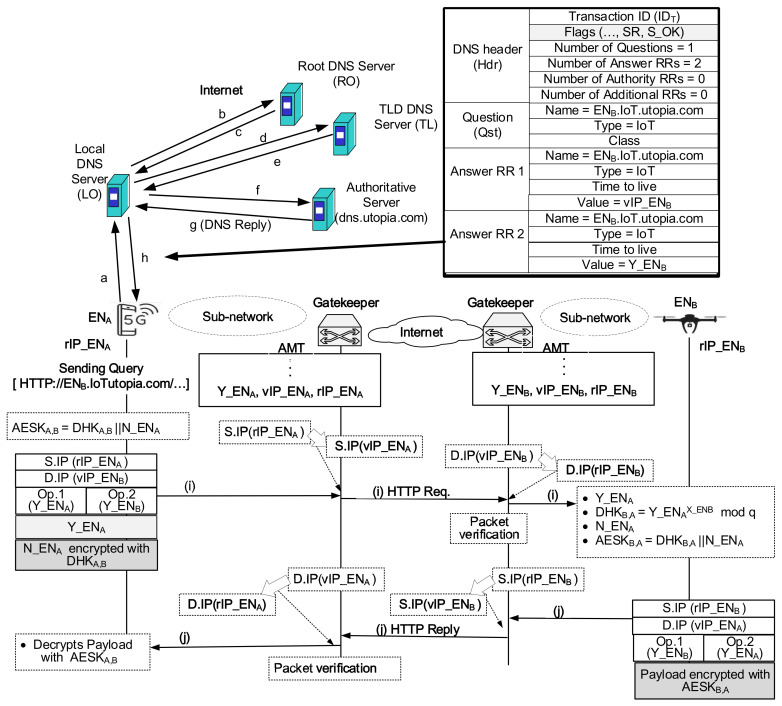
Secure HTTP communication over DNS-based Secure Gatekeeper System.

Figure 6 shows that the IoT device uses a symmetric key generated securely without using SSL to provide a secure HTTP Request/HTTP Reply communication via a DNS. Here, the calling device operates like a client, and the callee device acts as a server. If any device knows the callee device’s name, it can obtain callee’s name-address mapping information through the query/response to the DNS system. Let’s assume that the name of the calling device is $EN_{A}$. The $EN_{A}$-side gatekeeper maintains $EN_{A}$-related AMT entry of [public value = $Y_{{EN}_{A}}$, virtual IP address = $vIP\_EN_{A}$, real IP address = $rIP\_EN_{A}$]. Also, the name of the callee device is $EN_{B}$. The $EN_{B}$-side gatekeeper maintains $EN_{B}$-related AMT entry of [public value = $Y\_{EN}_{B}$, virtual IP address = $vIP\_EN_{B}$, real IP address = $rIP\_EN_{B}$]. The AU server maintains the Resource Record (RR) database, where an RR consists of [Name, Value, Type, TTL (Time-To-Live)]. Assuming that $EN_{B}$ belongs to the domain utopia.com, it’s Authoritative (AU) server stores two RRs: the first RR and second RR that contain [$EN_{B}$.IoT.utopia.com, $vIP\_EN_{B}$, IoT, TTL] and [$EN_{B}$.IoT.utopia.com, $Y\_{EN}_{B}$, IoT, TTL], respectively.

Below we enumerate steps to open secure peer-to-peer communication over DNS-based Secure Gatekeeper System.

- First, $EN_{A}$ sends the DNS query information of $EN_{B}$.IoT.utopia.com to the domain utopia.com Authoritative (AU) server, which belongs to $EN_{B}$.
- The AU server on the domain utopia.com that received it finds two RRs for $EN_{B}$ from the Resource Record (RR) database.
- The AU server creates the DNS reply containing this two RR information and sends it to $EN_{A}$.
- When $EN_{A}$ receives the DNS reply, it obtains the [public value = $Y\_{EN}_{B}$, virtual IP address = $vIP\_EN_{B}$] information from the DNS reply.
- Finally, $EN_{A}$ can send an HTTP Req toward $EN_{B}$ with [S.IP = $rIP\_EN_{A}$, D.IP = $vIP\_EN_{B}$, Op.1 = $Y\_{EN}_{A}$, Op.2 = $Y\_{EN}_{B}$]. Because ${EN}_{A}$ securely keeps its secret value ($X\_{EN}_{A}$) and nonce ($N\_{EN}_{A}$), it calculates the 16 bit-size DH key, that is, $DHK_{A,B}={Y\_{EN}_{B}}^{X\_{EN}_{A}}\;mod\;q$. And the concatenation process results in 128 bit-size AES key, that is, $AESK_{A,B}=DHK_{A,B}\left|\right|N\_{EN}_{A}$. The application header of the HTTP Req packet contains the public value in a clear form ($Y\_{EN}_{A}$) and $N\_{EN}_{A}$ encrypted with $DHK_{A,B}$.
- As the HTTP Req packet passes through the $EN_{A}$-side gatekeeper, it changes S.IP form $rIP\_EN_{A}$ to $vIP\_EN_{A}$.
- As the HTTP Req packet passes through the $EN_{B}$-side gatekeeper, it changes D.IP form $vIP\_EN_{B}$ to $rIP\_EN_{B}$. When the HTTP Req packet arrives at the $EN_{B}$-side gatekeeper, if $Y\_{EN}_{B}$ of Op.2 field is different from that of the original packet at the departure, the packet will fail to reach the final destination. It is because that wrong value of $Y\_{EN}_{B}$ hardly matches the proper $rIP\_EN_{B}$.
- When $EN_{B}$ receives the HTTP Req packet, it first extracts the public value $Y\_{EN}_{A}$ from the Op.1 field. Secondly, it extracts the corresponding value which its application header includes. Comparing these two values, $EN_{B}$ authenticates that the HTTP Req packet’s source is $EN_{A}$ for the case that these two values are the same. With the extracted public value of $Y\_{EN}_{A}$ and $EN_{B}$’s secret value of $X\_{EN}_{B}$, $EN_{B}$ calculates the symmetric key $DHK_{B,A}$ using the equation of ${Y\_EN_{A}}^{X\_{EN}_{B}}\;mod\;q$. And it decrypts the second part of the application header using $DHK_{B,A}$ and obtains $N\_{EN}_{A}$. Now, ${EN}_{B}$ can calculate its AES key $AESK_{B,A}$ by concatenating $DHK_{B,A}$ and $N\_{EN}_{A}$. As a result, ${EN}_{B}$ can decrypt the payload using $AESK_{B,A}$. Now, $EN_{B}$ can send an HTTP Reply toward $EN_{A}$ with [S.IP = $rIP\_EN_{B}$, D.IP = $vIP\_EN_{A}$, Op.1 = $Y\_{EN}_{B}$, Op.2 = $Y\_{EN}_{A}$]. Also, the payload of the HTTP Reply packet contains the contents encrypted with $AESK_{B,A}$. When the HTTP Reply arrives at $EN_{A}$, it can decrypt the payload using the secret key of $AESK_{A,B}$ where $AESK_{A,B}=DHK_{A,B}\|N\_{EN}_{A}$.

### 3.3. Secure Gatekeeper System for V2V, IoT2V, or V2IoT Communication

**Figure 7:**
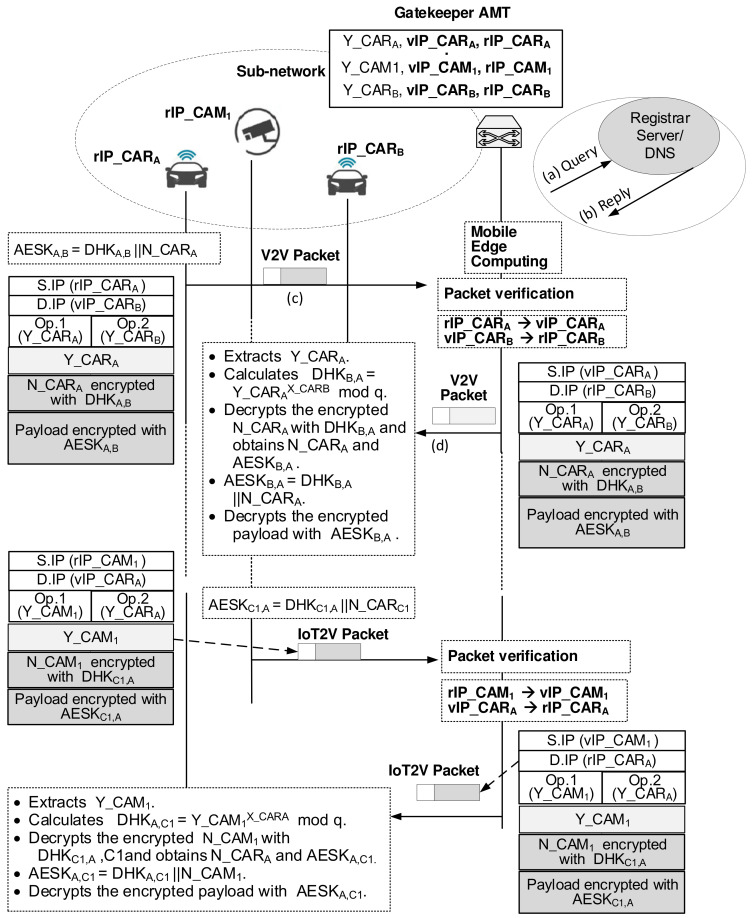
Gatekeeper-centric V2V and IoT2V communication.

Figure 7 describes the secure gatekeeper system that can apply to V2V and IoT2V communication. Let’s say that the Registrar Server or the DNS AU server already has the address-related information for the car A ($CAR_{A}$) and car B ($CAR_{B}$), which are behind the same sub-network. Then, the gatekeeper holds the entry of [$Y\_CAR_{A}$, $vIP\_CAR_{A}$, $rIP\_CAR_{A}$] for $CAR_{A}$ and [$Y\_CAR_{B}$, $vIP\_CAR_{B}$, $rIP\_CAR_{B}$] for $CAR_{B}$. When $CAR_{A}$ sends a V2V packet to the destination $CAR_{B}$, $CAR_{A}$ uses the Query/ Reply procedure to obtain the address-related information of [$Y\_CAR_{B}$, $vIP\_CAR_{B}$] via the Registrar Server or DNS system. When $CAR_{A}$ sends a V2V packet to $CAR_{B}$, it uses the value of $Y\_CAR_{B}$ obtained by the Query/Reply, and the secret value $X\_CAR_{A}$ that $CAR_{A}$ keeps securely to calculate symmetrical key $DHK_{A,B}$ using the function of ${Y\_CAR_{B}}^{X\_CAR_{A}}\;mod\;q$. ${CAR}_{A}$ generates a nonce ($N\_{CAR}_{A}$). Then, it produces 128 bit-size AES key, that is, $AESK_{A,B}\;=\;DHK_{A,B}\left|\right|N\_{CAR}_{A}$. When generating a V2V packet to be sent, the IP header’s relevant fields include [S.IP = $rIP\_CAR_{A}$, D.IP = $vIP\_CAR_{B}$, Op.1 = $Y\_CAR_{A}$, Op.2 = $Y\_CAR_{B}$]. The application header of the V2V packet contains the public value in a clear form ($Y\_{CAR}_{A}$) and $N\_{CAR}_{A}$ encrypted with $DHK_{A,B}$. The application layer payload of the V2V packet is encrypted using the symmetric key $AESK_{A,B}$ calculated earlier. Thus, the V2V packet travels toward the destination ${CAR}_{B}$. When the gatekeeper receives this packet, knowing that the final destination is in the same sub-network, the gatekeeper relays it to $CAR_{B}$ after the Op.2-based IP address conversion process from S.IP = $rIP\_CAR_{A}$ and D.IP = $vIP\_CAR_{B}$ to S.IP = $vIP\_CAR_{A}$ and D.IP = $rIP\_CAR_{B}$. Here, hackers’ attacks on the Op.2 public value is useless because the modified value of Op.2 public value certainly leads to AMT matching failure at the gatekeeper. When $CAR_{B}$ receives the encrypted V2V packet, it extracts the Op.1 public value, that is, $Y\_CAR_{A}$. Then, it calculates symmetrical key $DHK_{B,A}$ using the function of ${Y\_CAR_{A}}^{X\_CAR_{B}}\;mod\;q$. And it decrypts the second part of the application header using $DHK_{B,A}$ and obtains $N\_{CAR}_{A}$. By concatenating $DHK_{B,A}$ and $N\_{CAR}_{A}$, it can calculate its AES key $AESK_{B,A}$. As a result, it can decrypt the payload using $AESK_{B,A}$.

Second, Let’s consider the situation that the IoT camera 1 ($CAM_{1}$) sends an urgent information to car A ($CAR_{A}$) via real-time IoT-to-Vehicle communication. In advance, $CAM_{1}$ can obtain the address-related knowledge of [$Y\_CAR_{A}$, $vIP\_CAR_{A}$] via the Registrar Server or DNS system. So, in the urgent situation, $CAM_{1}$ can send the IoT2V packet without the Query/ Reply procedure. $CAM_{1}$ uses the public value $Y\_CAR_{A}$ and the secret value $X\_CAM_{1}$ to calculate symmetrical key $DHK_{C1,A}$ using the function of ${Y\_CAR_{A}}^{X\_CAM_{1}}\;mod\;q$. Following the same steps as the case of the previous V2V communication, $CAM_{1}$ produces the AES key ($AESK_{C1,A}=DHK_{C1,A}\left|\right|N\_{CAM}_{1}$). For the IoT2V packet to be sent, the IP header’s relevant fields include [S.IP = $rIP\_CAM_{1}$, D.IP = $vIP\_CAR_{A}$, Op.1 = $Y\_CAM_{1}$, Op.2 = $Y\_CAR_{A}$]. The application header of the IoT2V packet contains the public value in a clear form ($Y\_{CAM}_{1}$) and $N\_{CAM}_{1}$ encrypted with $DHK_{C1,A}$. The application layer payload of the IoT2V packet is encrypted using the symmetric key $AESK_{C1,A}$. When the gatekeeper receives the IoT2V packet, it relays the packet to $CAR_{A}$ after changing relevant IP header fields. When $CAR_{A}$ receives the encrypted IoT2V packet, in sequence, it extracts $Y\_CAM_{1}$, calculates symmetrical key $DHK_{A,C1}$, and produces its AES key $AESK_{A,C1}$. As a result, it can decrypt the payload using $AESK_{A,C1}$.

## 4. Improvement Effects of the Secure Gatekeeper System

### 4.1. Packet Verification

In the secure gatekeeper system, the EN, gatekeeper, and Registrar Server (or DNS system) share the end node’s DH public value. The Registrar Server or DNS intervenes to provide the session initiator and session responder the peer’s DH public value, which is already certified by them. Suppose the information of these three does not maintain consistency for a specific end node. In that case, other nodes will face traversal problems to fail to pass through that end node’s gatekeeper for reaching that end node correctly. Any node can securely obtain the other party’s DH public value through the Registrar Server (or DNS system). However, the session initiator should contain its DH public value in the sending packet’s IP header. When the session responder receives the packet, it verifies its validness through the Query/Reply mechanism to the Registrar Server (or DNS system). This paper does not assume the worst situation that even the hacker node maintains consistency on its DH public value among the hacker node, its gatekeeper, and the Registrar Server (or DNS system).

For the session initiator node ($EN_{S}$) and the session responder node ($EN_{D}$), when the packet with [Op.1 = $Y\_{EN}_{S}$] and [Op.2 = $Y\_{EN}_{D}$], which departs from $EN_{S}$, arrives via the Internet at the gatekeeper belonging to $EN_{D}$, the gatekeeper examines the packet’s header field of [Op.2 = $Y\_{EN}_{D}$] and finds the actual IP address of the destination node. Here, an external hacker cannot modify the public value of $Y\_{EN}_{D}$. If $Y\_{EN}_{D}$ of Op.2 field is different from that of the original packet at the departure, the packet will fail to reach the final destination. It affects that the gatekeeper blocks the enter of uncertified packets because the wrong value of $Y\_{EN}_{D}$ hardly matches the proper $rIP\_EN_{D}$. So, the actual packet verification process occurs at the entry point of the destination gatekeeper. The following scenario is related to the case that $EN_{S}$ is the hacker node itself ($EN_{H}$). When the packet with [Op.1 = $Y\_{EN}_{H}$] and [Op.2 = $Y\_{EN}_{D}$], which departs from $EN_{H}$, arrives at the $EN_{H}$-side gatekeeper, the gatekeeper examines the packet’s header field of [Op.1 = $Y\_{EN}_{H}$]. Here, the gatekeeper will fail to find the hacker node’s virtual IP address because it is difficult for the hacker node to maintain consistency on its DH public value together with the gatekeeper and the Registrar Server (or DNS system). Then, the gatekeeper will drop the packet having the invalid Op.1 field value.

### 4.2. Authentication

**Figure 8:**
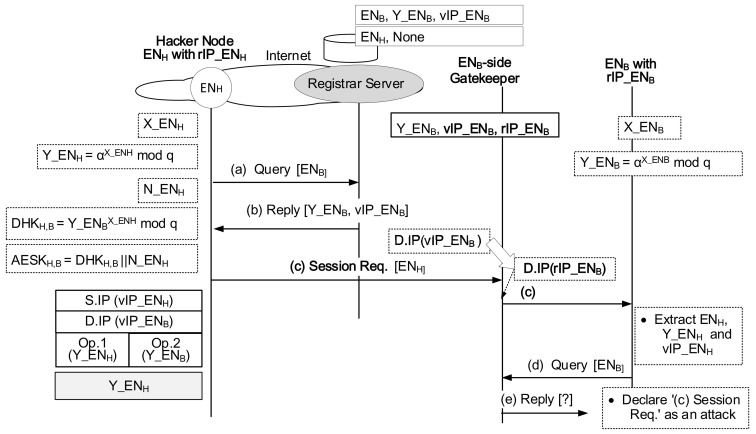
Effectiveness of the secure gatekeeper system against malicious session requests.

In the secure gatekeeper system, the Registrar Server or DNS intervenes to provide the session initiator the peer’s DH public value, which is certified by them. The Registrar Server or DNS keeps the session initiator’s DH public value only for which it certifies. Then, the session responder can decide whether it accepts the session request or not. In the secure gatekeeper system, the Registrar Server or DNS functions as an authentication server that provides an authentication service to the session responder. The session-responder IoT authenticates the client IoT approaching it with the help of them. Let’s assume the attack scenario that the hacker node ($EN_{H}$) sends the malicious session request packet to the IoT node ($EN_{B}$). The packet will pass the $EN_{B}$-side gatekeeper because even the hacker can access $EN_{B}$’s public information [$Y\_{EN}_{B}$, $vIP\_EN_{B}$] in the database of the registrar server. However, When the packet arrives at the destination node ($EN_{B}$), $EN_{B}$ extracts the identification $EN_{H}$ from the payload of the packet and examines the packet’s header field of [Op.1 = $Y\_{EN}_{H}$]. Here, $EN_{B}$ can add the authentication procedure to check whether $EN_{H}$ is legitimate by asking it to the registrar server ((d) and (e) in Figure 8). Because the registrar server has the certified public values for all IoTs behind the distributed gatekeepers, $EN_{B}$ will declare the session trial as ’Unauthenticated’ if (e) Reply is incorrect. Then, $EN_{B}$ rejects the session request from the $EN_{H}$. As a result, any IoT device can try to reach a certain peer IoT because the peer’s DH public value is made public via the Registrar Server or DNS. However, the secure session is successfully established under the fair condition that the initiator’s DH public value is declared valid via the Query/Reply procedure of the peer IoT.

### 4.3. Defending against Traffic Analysis Attacks

The gatekeeper allocates the matched pair of real IP address and virtual IP address to each end node behind it. For the outgoing and incoming datagrams, the gatekeeper performs rIP-vIP and vIP-rIP translations. When two peers exchange signaling messages to set up a session, they can hide their identities or real IP addresses. During the data transfer period, virtual addresses seen by outside have no relationship with corresponding actual IP addresses. As long as the DH public value is trustworthy, our secure gatekeeper system contributes to defending against traffic analysis attacks.

For the IoT connections, traffic usually carries small-sized control messages. Traffic analysis attacks cannot but infer the limited information of the IP address and name of the IoT device. Hence, our SGS proposed is almost safe against traffic analysis attacks.

Table 2 shows signaling parameters visible to the attackers in three different cases: RS SGS shown in Figure 5, DNS SGS shown in Figure 6, and MEC SGS shown in Figure 7. There is no risk of exposing essential parameters during the signaling phase. Considering that only the blind value of [Y_EN] in the application payload is exposed to the enemy, the enemy rarely seizes opportunities to identify and attack a specific device.

### 4.4. Reliability

Suppose sensors in autonomous vehicles and nearby IoT cameras communicate through L2 connections. In that case, the received data’s reliability cannot guarantee because, typically, IoT devices do not have enough computing power to determine whether malicious packets are coming. Meanwhile, because the examination action occurs on the cross-gatekeeper basis, the gatekeeper performs to protect the malicious packets’ intrusion in advance. Thus, sensors in autonomous vehicles and nearby IoT cameras can safely communicate with each other with trust without the burden of detecting malicious packets.

### 4.5. Confidentiality

There is a concern about MITM attacks against authentication when exchanging DH public values in the DH key exchange between the two devices. However, in our secure gatekeeper system, the end node, gatekeeper, and Registrar Server (or DNS) maintain consistency on the DH public values, enabling two end-to-end peers to involve in the regulated DH parameter exchanges preventing from the enemy’s attacks on the shared key generation. With the extracted DH public value sent from the authenticated source node and self-keeping secret value, the destination node calculates the DH symmetric key. However, this DH shared key between two peers will be the same for their different sessions. So, the secure gatekeeper system requires to creates a one-time session key for the individual session even for the same peers. The session initiating node creates a nonce number on a session basis. Another purpose of using the nonce value is to adjust the size of the final symmetric key to 128 bits that can apply to the Advanced Encryption System (AES). That is why the size of the nonce value is 112 bits, while the size of the DH key is 16 bits. The nonce is securely delivered to the destination node together with the DH public value of the source node. So, the end-to-end peers will share 128 bit-size AES key. Therefore, the data packet’s payload contains the contents encrypted with the AES symmetric key, which is the one-time session key. A brute force attack has to involve trying every possible key until the decryption is successful. Considering that our secure gatekeeper system uses a 128 bit-size one-time session key, the attacker needs to work half of all possible keys: $2^{127}$ to achieve success. Therefore, our secure gatekeeper system satisfies confidentiality services even against the brute force attack.

### 4.6. Security Performance

Asymmetric algorithms are complex in executing and utilizing more resources because of 1024-bit key pairs of a private key and public key than symmetric algorithms. Thus, most IoT networks use symmetric algorithms because they are easy to implement, utilize fewer resources with low overhead, and are faster. However, it had only one weak point related to the authentication issue during the shared key agreement procedure. Table 3 shows the level of security performance our SGS can provide. Diffie-Hellman key exchange requires modular exponential computation, that is, ${Y\_EN_{A}}^{X\_{EN}_{B}}\;mod\;q$ for $EN_{A}$ side, which is computationally inexpensive. Furthermore, all the integers used in this computation have the size of 16 bits. So, the DH key computation imposes a light computational load to low-end IoT devices. Meanwhile, they can produce a 128-bit final shared key by virtue of 112-bit one-time nonce, which requires a small amount of computational load to generate. The resultant 128-bit one-time shared key assures to provide a level of security similar to AES. Our SGS gives a solution for the DH key exchange’s weak point because of the gatekeeper-made authentication scheme that the gatekeeper involves authenticating peers. Furthermore, the gatekeeper verifies all the packets from entering, detects malicious packets, and prevent them from entering.

The virtual IP address technique applies in two ways: the MTD technique and the load balancing technique in a massive server farm. The MTD technique usually aims to defend against network reconnaissance and scanning attacks using a random virtual address for latency-sensitive applications such as vehicular communication. On the other hand, the virtual IP address is useful so that server load balancers can scale easily to meet a massive server farm’s demands as the amount of traffic and the number of servers in the server farm grows. Server load balancers can be scalable if the incoming data traffic to the gateway router at the data center is forwarded according to the assigned virtual IP address. When a request arrives at this collection of servers, or server farms, the request is routed to a particular server based on the virtual addresses. The multiple requests that come at the server farm should be distributed among the various servers. In this case, the performance analysis should include the metric in the load balancers, explaining how it scales easily the multiples virtual addresses as multiple requests increase.

For the gatekeeper to operate correctly, a real IP-virtual IP conversion occurs in real-time for every passing packet. In addition to modifying the IP address, the gatekeeper must change the IP checksum. These two workloads characterize the scalability of the gatekeeper, which supports a large number of devices for a given domain. Considering that our gatekeepers are deployed in a distributed manner and deal with P2P applications, the gatekeeper’s scalability is clarified different from Firewalls located at the big web-based shopping center entrance. Our secure gatekeeper system needs the performance metric to describe the latency issue rather than the scalability issue.

### 4.7. Latency Performance

The following analysis relates to the communication latency in three different cases: RS SGS, DNS SGS, and MEC SGS. Each system causes two kinds of latency: Signaling latency, which takes for a secure session establishment, and data transfer latency required to transfer a data packet from the source to the destination. This paper assumes three components that cause latency:

- $L_{I}$: intra-domain path latency caused in intra-domain,
- $L_{II}$: latency caused in the end-to-end path,
- $L_{III}$: latency caused to collaborate with the distributed servers, which are spread in inter-domain regions,

where $L_{II}$ = 5$L_{I}$, and $L_{III}$ = 10$L_{I}$. This assumption is based on the TraceRT utility program’s test data to measure pure round trip network delays. For the Google.com site destination, the TraceRT tool’s test data shows the round trip delay of at most 6 ms within four hops and at least 30 ms within ten hops. Because the TraceRT program carries no application payload, this paper only introduces the ratio of two different types of measured delays for the SGS network architecture. Then, the unit delay of $L_{I}$ corresponds to the packet delay to travel from a certain end node to its gatekeeper, and the end-to-end path between two peers is longer by five times compared to the unit delay. In practice, the DNS response time test reveals the response time delay to the DNS query [39]. In this study, the response time is 62 ms. Another ICMP echo/reply test shows the round-trip average time of 85 ms. $L_{III}$ corresponds to the network delay of the Query/Reply mechanism plus searching delay in the Registra Server or DNS. Based on these results, this paper assumes that $L_{III}$ is twice as large compared to the end-to-end path delay. Samsung has announced that it has achieved 7.5 Gbps, the fastest ever 5G data transmission rate in a stationary environment. The company has achieved a stable connection at 1.2 Gbps in a mobile environment from a vehicle at a speed of 100 km/h at 28 GHz [40]. Considering that the existing 4G network shows the data rate of 100 Mbps and $L_{I}$-type latency of around 50 ms, $L_{I}$-type latency could drop up to 5 ms with 5G technology. This paper assumes that the unit delay of $L_{I}$ is going to be about 10 ms.

As shown in Table 4, the signaling latency to set up a secure session requires 120 ms in the RS SGS. DNS SGS and MEC SGS show similar latency levels of 200 ms and 120 ms, respectively. Data transfer latency needed to transfer a data packet from the source to the destination requires up to 50 ms for all systems.

**Figure 9:**
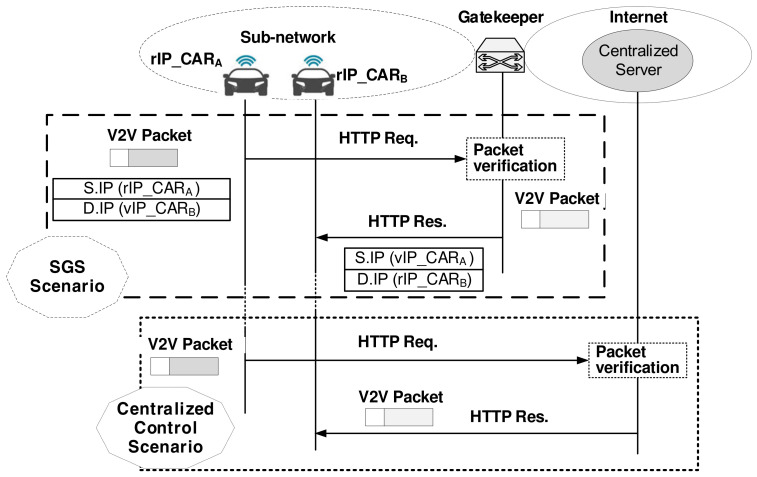
Performance measurements with the SGS testbed.

Ensuring the reliability of the in/out messages for the IoT device of limited capacity is possible if the server as a third party involves confirming the message’s maliciousness. The third-party services can be done by the gatekeeper proposed in this paper or the centralized server, located somewhere on the Internet. This paper suggests using the gatekeeper at the local network entrance, which acts as the third-party server. We built a testbed to obtain real performance data. In our testbed shown in Figure 9, the gatekeeper’s subnet link operates over Wi-Fi with a data rate of 72 Mbps. Our testbed runs a web’s application where $CAR_{A}$ sends an HTTP request, and $CAR_{B}$ receives the HTTP response and displays the replied Web object. There are two types of servers, which send the Web object: Gatekeeper-playing Web server that acts based on the SGS scenario and the centralized server that works based on the centralized control scenario.

IoT messages must be small enough to fit into their link-layer packets [41].

- It should fit within a single IP packet to avoid IP fragmentation (MTU of 1280 bytes for IPv6). If one considers the size of the headers, the acceptable upper bound is 1024 bytes for the payload size.
- Or less to avoid adaptation layer fragmentation (60–80 bytes for 6LoWPAN).

Suppose an IoT device needs to transfer larger payloads, which require to handle multiple blocks of information. We use four types of messages with different payload sizes: 3 Kbytes, 30 Kbytes, 300 Kbytes, and 3000 Kbytes. Each message emulates the following conditions.

- 3 KB-payload: [3 blocks over IPv6 or 37 blocks over 6LoWPAN],
- 30 KB-payload: [30 blocks over IPv6 or 370 blocks over 6LoWPAN],
- 300 KB-payload: [300 blocks over IPv6 or 3700 blocks over 6LoWPAN], and
- 3000 KB-payload: [3000 blocks over IPv6 or 37,000 blocks over 6LoWPAN].

So, the gatekeeper-playing server and the centralized server store 4 JPEG images with different sizes of 3 Kbytes, 30 Kbytes, 300 Kbytes, and 3000 Kbytes. As clients in the testbed, two cars use the same CPU core clock of 1.2 GHz and Android version 5.0.2. The gatekeeper-playing web server operates with a CPU core of 2.90 GHz. The testbed uses the external server supported by a Web hosting service company to provide enough private web service. We repeat HTTP sessions 30 times to obtain each latency data, that is, the delay from the departure of the HTTP request to the HTTP response’s arrival. To do so, we intentionally processed the speed of the experiment as late as possible. The experiment’s interval was intended to be about 30 min to exclude the effects of web caching on the server and the network’s flow recognition.

**Figure 10:**
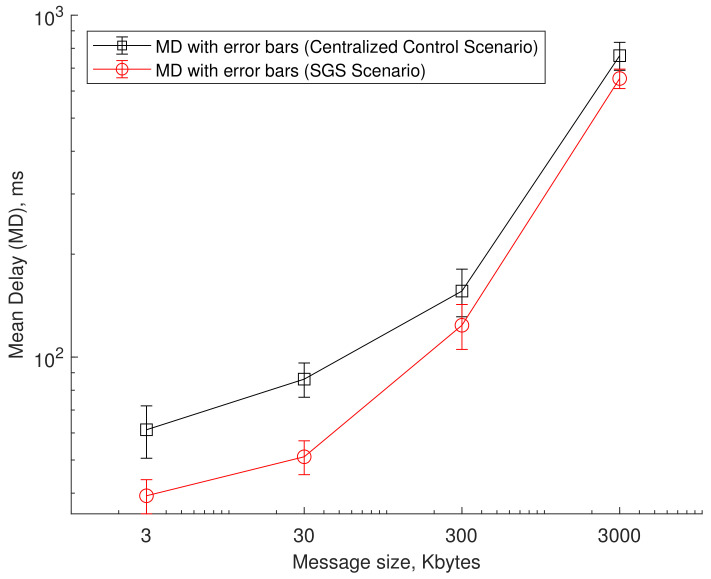
Latency comparisons between centralized control and SGS scenarios.

Figure 10 shows the experimental data that explains how long it takes for two different IoT devices to exchange one-time reliable data with each other with the help of a gatekeeper or the centralized server. It is shown that as the IoT message size increases, the latency increases. It reaches almost 100 ms for the message size of 30 Kbytes under the centralized control scenario environment. Also, the SGS scenario shows less variation on latency than Centralized Control Scenario. Under the condition that the gatekeeper’s subnet link operates over Wi-Fi with a data rate of 72 Mbps, the latency level in the SGS scenario meets the requirement that V2IoT communication should satisfy the latency level of 20 ms if the message size remains within 30 Kbytes.

**Figure 11:**
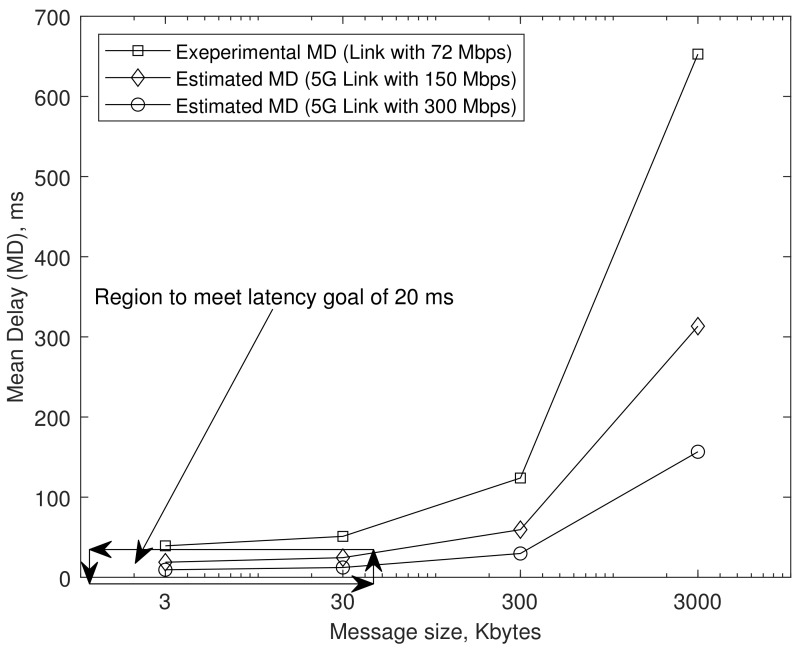
Estimated communication latency under the SGS scenario in 5G links.

Figure 11 explains how the latency goal of 20 ms can be achieved when our SGS operates under the environment of 5G links. Considering that the testbed’s subnet link operates with a data rate of 72 Mbps, the 5G link speed can increase to about 300 Mbps soon (by 30 April 2020, the user’s average 5G download speed already reaches the level of almost 500 Mbps for the case of Verizon in the USA). Even more positive is that the IoT message’s size can be limited to within the range of 30 Kbytes. It is shown that even in the 5G links, the latency increases rapidly as the message size exceeds 300 Kbytes. As discussed in Section 1, if IoT2V data transfer latency is limited within 20 ms, a car driving through the city at 60 km/hour can successfully take the reaction within a moving distance of about 0.3 meters away at the emergent situation. The region surrounded by arrow lines in Figure 11 meets our latency goal of 20 ms. As a result, our SGS can be a solution suitable for V2IoT communication to meet the latency condition of 20 ms if our SGS operates under the following conditions.

- Subnet link speed provides at least 150 Mbps.
- Message size is limited within 30 Kbytes.

### 4.8. Scalability

The gatekeeper covers all passing packets incoming and outgoing from the IoT devices located behind it. So, the number of devices for a given domain corresponds to the size of AMT. Considering that a real IP-virtual IP conversion occurs in real-time for every passing packet, the processing delay looking for AMT entry for a given blind value increases as the size of AMT increases. Then, the impact in performance regarding the size of AMT is related to find whether to complete lookup processing is done at incoming packet speed. Usually, the incoming packet speed is proportional to the number of devices for a given domain under the assumption that each IoT device sends messages with the same sending rate. This scalability analysis belongs to this paper’s further study area. However, In normal routers, the processing delay necessary for IP forwarding is less than several milliseconds. So, it is almost certainly estimated that in the gatekeeper system, latency’s main cause is due to all transmission delays along with the links of the communication path rather than the processing delay for address conversion inside the gatekeeper. The demonstration showed that the Cisco ASR9000 router performs that it can forward real traffic at 20 Gbps or higher [42]. These experimental results demonstrate the gatekeeper can handle $\frac{20\times 10^{9}}{8\times 30\times 10^{3}}≅$ 83,000 30 KB-sized messages per second.

## 5. Conclusions

Existing approaches to use the dual use of the real and virtual addresses includes the NAT system, Moving Target Defense (MTD) technique, and the load balancing technique in a massive server farm. This paper suggests using the dual addresses of the virtual IP and real IP where the proposed gatekeeper plays the leading role in enabling the real IP addresses of IoTs inside local subnetworks to be not explicitly addressable and visible from outside of the gatekeeper. The gatekeeper shares the address mapping information with the registration server or DNS. While the registration server or DNS has global address mapping information, the gatekeeper maintains the mapping information for the local IoT devices. Also, the gatekeeper shares the information necessary for security management with the registration server or DNS. The gatekeeper plays a vital role in establishing end-to-end secure sessions between two IoTs located behind their gatekeepers (inter-gatekeeper sessions). Security concerns regarding the confidentiality and authentication of the peers’ communication are solved using some gatekeeper-made security scheme. The gatekeeper also leads edge computing for intra-gatekeeper communication for its own IoT devices. Our gatekeeper at the local network entrance acts as the third-party server. Then, the communication time delay, which occurs in Vehicle-to-IoT communication via a gatekeeper system, can be similar to the direct device-to-device (D2D) communication level. This paper suggested two inter-gatekeeper communication approaches (Registrar Server-based Secure Gatekeeper System and DNS-based Secure Gatekeeper System) and an intra-gatekeeper communication system (MEC Secure Gatekeeper System) focusing on reducing Vehicle-to-IoT communication satisfies the latency required to the level of 20 ms. Our analysis results showed that latency goal of 20 ms could be achieved when our MEC Secure Gatekeeper System operates under the environment of (i) subnet link speed with at least 150 Mbps, and (ii) message size limited within 30 Kbytes. Our further study area includes scalability analysis to find the impact on latency performance regarding the size of the address mapping table, the number of IoT devices for a given domain where the same gatekeeper covers.

## Figures and Tables

**Table 1:** Three different operational modes of the secure gatekeeper system.

Operational Modes	Types of Secure Sessions	Main Characteristics	Trusted Third Party
RS-based Secure Gatekeeper System	Inter-Gatekeeper Secure Connections	Gatekeeper-based Real IP/Virtual IP Conversion	Registrar Server involved
DNS-based Secure Gatekeeper System	Inter-Gatekeeper Secure Connections	Gatekeeper-based Real IP/Virtual IP Conversion	DNS Server involved
MEC-based Secure Gatekeeper System	Intra-Gatekeeper Secure Connections	Gatekeeper-based Real IP/Virtual IP Conversion and Low latency	DNS Server or Registrar Server involved

**Table 2:** Information exposed to hackers.

**RS SGS** (Figure 5)	[$Y\_{EN}_{A}$] in (c)
**DNS SGS** (Figure 6)	[$Y\_{EN}_{A}$] in (i)
**MEC SGS** (Figure 7)	[$Y\_{CAR}_{A}$] in (a) and (b)

**Table 3:** Security performance.

	Security Management	Key Size (bits)	Security Performance
One-time	DH key computation	16	Light computational load
Shared Key	One-time Nonce	112	Small computational load
Agreement	Final shared key	128	Use of possible key length up to 128 bits
Authentication			Gatekeeper involved Registrar Server (or DNS) involved
Intrusion detection			Gatekeeper involved

**Table 4:** Latency comparisons.

		Delay Components	Latency (Milliseconds)
**RS SGS** (Figure 5)	Signaling	$L_{I}$: (a), (b), $L_{II}$: (c), (d) $\left(2L_{I}+2L_{II}\right)$	120
	Data	$L_{II}$: (g)	50
**DNS SGS** (Figure 6)	Signaling	$L_{II}$: (i), (j), $L_{III}$: (a, b, ⋯, h) $\left(2L_{II}+L_{III}\right)$	200
	Data	$L_{II}$: (j)	50
**MEC SGS** (Figure 7)	Signaling	$L_{III}$: (a,b), $L_{I}$: (c), (d) $\left(L_{III}+2L_{I}\right)$	120
	Data	$L_{I}$: (c), (d)	20

## Abbreviations

The following abbreviations are used in this manuscript:

SGS	Secure Gatekeeper System
IoT	Internet of Things
NAT	Network Address Translation
MTD	Moving Target Defense
SDN	Software-Defined Networking
ECU	Electronic Control Unit
AMT	Address Mapping Table
SSL	Secure Socket Layer
TLS	Transport Layer Security
DNS	Domain Name System
IPSec	Internet Protocol Security
VPN	virtual private network
IP	Internet Protocol
V2V	Vehicle-to-Vehicle
DH	Diffie-Hellman
D2D	device-to-device
MITM	man-in-the-middle
SIP	Session Initiation Protocol
AU	AUthoritative (AU)
IoT2V	IoT-to-Vehicle
V2IoT	Vehicle-to-IoT
EN	end nodes
P2P	Peer-to-Peer
MEC	Mobile Edge Computing
AES	Advanced Encryption System
LwM2M	Lightweight Machine-to-Machine
MTU	Maximum Transmission Unit
6LoWPAN	Low power Wireless Personal Area Network
CoAP	Constrained Application Protocol
